# Slaughter-House Horrors

**Published:** 1923-07

**Authors:** Nina Hamilton


### CORRESPONDENCE.

[Correspondence on all subjects is invited, but we cannot be responsible for the opinions expressed by our correspondents, who must give name and address as a guarantee of good faith, but not necessarily for publication. Correspondents are reminded that conciseness greatly facilitates early insertion.]

### Slaughter-house Horrors.

*To the Editor of* The Hospital and Health Review.

Sir,—Last July I had my first experience of the interior of a slaughter-house, namely, London's largest slaughter-house, at Islington—which is under the direction of the City of London. There was a certain amount of difficulty in being admitted, so different from the treatment accorded to visitors to the public abattoirs of Berne, Basle, Stockholm and other cities abroad, where the slaughter-house system is well managed, with inspection both with regard to the humane treatment of animals, and the cleanly after-treatment of the meat. Eventually, through the courtesy of the Superintendent, who made it quite plain that it was an act of courtesy on his part and not our right as citizens, we were shown in. I asked to see the animals being pole-axed, but, to my surprise, none were being killed by that method. Only the Jewish method was being employed. We have since been informed that 75 per cent. of all cattle killed at Islington are killed by the Jewish method. We were then taken to a most horrible scene.

Picture a three-walled booth with a door at the back for the entry of the animals, the floor covered with blood, the carcases of the freshly slain hanging up, and, horror of horrors! a living creature waiting its doom amid all the carnage. I said to the Superintendent, "Is it possible that you have a living animal among all this?" "Oh," he replied, "its head is turned the other way." But it had to be brought in facing all the blood and carcases, and blood was all around.

I then saw the whole Jewish procedure. The poor creature, which was standing tied to the wall, had chains fastened round its legs. The chains were worked by a lever, which jerked the bullock off its feet, and it fell violently on to the hard cement floor. The head was then forced back into a very strained position by means of a lever and bar placed in the mouth. When this had been done, the Jewish slaughterman (*shochet*) was called. He came, and with a very sharp knife cut across the throat. The head was then released, and the animal, fully conscious, because the spinal cord was uninjured, slowly bled to death. The *shochet* came to me, and took much trouble to explain that since the knife was so sharp there could be no pain. He had cut his finger with it one day, it seemed, and it had not hurt him. I was struck by the childish comparison. For how could there be similarity between a slight cut on one's finger and cutting all the sensitive nerves, arteries, blood-vessels, etc., in the neck? If the sharpness of a knife really obliterated pain, where would be the necessity for anæsthesia in a surgical operation?

I saw four bullocks slowly ebb their lives out in this way. It was a piteous sight, for they were obviously conscious, and one poor beast lay moaning. It is quite false to say, as Jews have said to me, that the movements are only reflex. I have seen many animals stunned with the humane-killer and pole-axed, and in each case the movements were quite different. British butchers have told me that whenever possible they sever the spinal cord when the *shochet* has gone to wash his knife. One butcher said he pole-axed a bull as it lay on the floor, so greatly was he touched by its agony.

Since that time I have seen many slaughter-houses and have witnessed the usual methods of pole-axing and killing with the knife alone. These present prevalent modes of slaughter leave much to be desired. The barbarous idea that pigs must be hung up alive and then bled to death, otherwise their flesh is not dainty enough for our palates, is only comparable to the primitive outlook of the savage cannibal tribes, who, as Mr. Ward relates in his book "A Voice from the Cogo," consider their wretched human victims only sufficiently succulent for their palates if they have been immersed in water for some hours before they are killed.

But I think that to witness the killing of sheep by knife alone is almost the most heart-rending of all. One is reminded of "He is brought as a lamb to the slaughter, and as a sheep before her shearers is dumb, so He openeth not His mouth" (Isaiah, 53, vii.). They utter no sound, there is only the dumb, terrified look in their eyes, and their helpless struggles to get up from the crutch on which they are fastened as they bleed to death. They are supposed to have their necks broken directly after the throat is cut, and when this is done skilfully the release of death comes more quickly, but I have often seen this not properly carried out, and then minutes elapse before their sufferings are over.

This prolongation of the final throes of death is utterly unnecessary, for again and again I have witnessed the instant unconsciousness that supervenes with every kind of animal when a humane-killer is properly used. The proper employment of these instruments is so easy that a child can use them with safety, except that one does not like childhood to participate in slaughter.

From the hygienic and sanitary point of view, what can be worse than the pain-poisoned meat which is the penalty dealt to man as the result of the terror and anguish suffered by the animal when brought to a death of uncertain swiftness from pole-axe or knife, in a foul and blood-stained slaughterhouse? I have personally witnessed the killing in slaughter-houses in London, Carlisle, Glasgow, and Edinburgh. It seems to me a great disgrace that London, the largest and most important of these big cities, has the worst arrangements of all.

The Jewish method, so largely practised at Islington, was utterly condemned by the Admiralty Report of 1904, and is considered so cruel by the Swiss that it is forbidden in their country. We must realise that this is essentially a question which does not merely concern Jews, for the Jews only eat a part of the animal they pass as "Kosher," and none of the many animals which, after being killed by the Jewish method, they reject as being unfit for Jews. The unknowing Christian eats the rest of this painfully procured meat. Therefore the subject vitally concerns us all. Carlisle is a shining example to London in that there, under the kindly direction of Mr. John Dodds, Superintendent and Meat Inspector of the Carlisle Public Slaughterhouse, cattle have been humanely killed for 23 years with no difficulty at all. Glasgow has magnificent airy buildings, the best I have seen in these Islands, and the meat is dealt with in a clean and efficient manner, but so far, humane killing is not universal, though there is hope for the future from the influence of the enlightened Baillie Brechin, whose firm has adopted the humane killer for some months.

From every point of view a well-designed and properly-managed public abattoir is infinitely superior to the small and often dark and dirty premises of a private slaughterhouse. Is it not contrary to all reason that any place in which the food of the people is prepared should be considered "private" and remain without proper inspection, especially when that food is procured from living sentient creatures, which are liable to the ills that unhygienic civilisation brings in its train? Think of the difference between meat left lying on a floor which is not washed down after each batch of slaughtered animals, and that prepared in an airy, adequate building, where each carcass in turn is hauled up and removed suspended on runners. Again, in all the public abattoirs that I have seen there is always a special department for diseased meat; in a private slaughterhouse there can be no such provision.

I make no charge of cruelty or any other complaint against butchers; they are much the same as members of any other profession, just human and liable to temptation; but I have been shown how easy it would be to scrape off the tell-tale excrescences on a tuberculous carcass and sell it as ordinary meat, and such a temptation would be very hard to withstand when any pecuniary loss would entail suffering on wife and children. In the Swedish abattoirs each butcher insures his beast, and if it is diseased he receives full value and suffers no loss, and the general public is saved from eating diseased meat. Is it not time for our would-be spiritual leaders, the clergy, and our physical advisers, the doctors, to unite in endeavouring to abolish the barbarous cruelty of our present system, and the prevailing dirty and unhygienic methods of dealing with this important part of the people's food?

Nina Hamilton and Brandon.

